# The microwave-absorption properties and mechanism of phenyl silicone rubber/CIPs/graphene composites after thermal-aging in an elevated temperature

**DOI:** 10.1038/s41598-022-08415-6

**Published:** 2022-03-14

**Authors:** Xiao Yan, Jianhua Guo, Xinghua Jiang

**Affiliations:** 1 School of Materials Science and Engineering, South China University of Technology, Guangzhou, 510640 China; 2 School of Mechanical and Automotive Engineering, South China University of Technology, Guangazhou, 510640 China; 3 Zhongshan Institute of Modern Industrial Technology of SCUT, Zhongshan, 528400 China

**Keywords:** Materials science, Nanoscale materials, Structural materials

## Abstract

Recently, the application and development of flexible microwave-absorption composites based on silicone rubber have gradually become a research hot spot. In this study, methyl vinyl phenyl silicone rubber (MPVQ)/carbonyl iron particles (CIPs)/graphene (GR) composites were prepared by mechanical blending, and the effects of thermal-ageing temperature on the microwave-absorption properties of the composites were investigated. The mechanism of the thermal-ageing temperature’s effects on microwave-absorption behaviour was identified. The results show that unaged composites have superior microwave-absorption properties, with a minimum reflection loss (*RL*_*min*_) of − 87.73 dB, a lowest thickness of 1.46 mm, and an effective absorption bandwidth (*EAB*, *RL* < − 10 dB) reaching 5.8 GHz (9.9–15.7 GHz). With ageing at 240 °C for 24 h, the *RL*_*min*_ at a frequency of 5.48 GHz is − 45.55 dB with a thickness of 2.55 mm, and the *EAB* value reaches 2 GHz (range 4.6–6.6 GHz). In the thermal-ageing process, a crosslinking reaction occurs in MPVQ with an increase in crosslinking density from 5.88 × 10^−5^ mol g^−1^ (unaged) to 4.69 × 10^−4^ mol g^−1^ (aged at 240 °C). Simultaneously, thermal degradation of the composites leads to a reduction in the rubber concentration. In addition, a small amount of CIPs are oxidized to Fe_3_O_4_, and the remaining CIPs aggregate to generate more electrically conductive pathways. Consequently, the dielectric loss of the composites will be significantly improved, resulting in poor impedance matching. The microwave-absorption properties of the composites gradually decrease with increasing thermal-ageing temperature from 200 to 240 °C.

## Introduction

In recent years, with the prosperous development of the electronic communication industry, the issue of electromagnetic wave pollution has become increasingly serious, which is harmful to human health, information safety and the ecological environment^1–5^. Therefore, research and development to produce thin, broad-bandwidth and low-cost microwave-absorption materials with strong absorption abilities are the primary objectives of this industry^6–8^. Among available microwave-absorption materials, some based on a rubber matrix have not only superior microwave-absorption properties but also excellent mechanical and processing performance, thus attracting considerable attention^9,10^. Most rubbers are electromagnetic-wave transparent, which has a minor influence on microwave-absorption properties. To prepare high-performance rubber-based absorbing materials, some wave-absorbing fillers are essential in the rubber matrix, such as dielectric materials (graphite, graphene, carbon nanotubes and so on) and magnetic materials, including CIPs, FeSiAl, ferrite and so on^11–13^. For example, Xun et al.^14^ prepared carbonyl iron powder (CIP)/multi-walled carbon nanotubes (MWCNTs)/silicone rubber (VMQ) composites exhibiting excellent microwave-absorption performance with a minimum reflection loss (*RL*_*min*_) of − 37.5 dB and an effective absorption bandwidth (*EAB*) of 3.7 GHz. CIP/polyurethane (PU)/graphene (GR) composites were manufactured by Duan et al.^15^, who demonstrated that the synergistic effects of dielectric/magnetic composites can improve electromagnetic wave attenuation. However, when thermal pyrolysis or excessive crosslinking of some rubbers occurs at an elevated temperature, the concentration and distribution of the microwave-absorption fillers in the rubber matrix will be varied and further influence the microwave-absorption properties of the materials. In addition, destruction of the molecular structures of the rubber matrix will reduce their protection of the microwave-absorption fillers, resulting in high-temperature oxidation of the fillers^16^. For example, Zhang et al.^17^ reported the effect of thermal-ageing time on the microwave-absorption properties of CIP/VMQ composites. After ageing at 200 °C for 12 days, the *RL*_*min*_ of the composites changed from − 33.7 to − 18.8 dB, and the *EAB* decreased from 1.76 to 1.02 GHz, indicating that the thermal-ageing resistance of silicone rubber as the matrix needs to be further improved, while using only CIPs as microwave absorbers has disadvantages in balancing the complex relative permittivity and permeability. Notably, a hybrid of CIPs and GR with a relatively low density and high electrical conductivity is beneficial for improving impedance matching and the microwave-absorption performance of the composites^18–21^.

Herein, methyl vinyl phenyl silicone rubber (MPVQ) with better thermal stability was applied as the matrix accompanied by CIPs and GR as the microwave-absorption fillers. The effects of the thermal-ageing temperature on the microwave-absorption properties, mechanical properties and microstructure of the composites were investigated. Moreover, the microwave-absorption mechanism of the composites after thermal ageing was identified.

## Experimental section

### Materials

MPVQ with a phenyl content of 20% and a vinyl content of 0.4%) was purchased from Shandong Zhaogui Polymer Material Technology Co. Ltd., Shandong, China. CIPs with an average particle size of 4 μm were produced by Shenzhen Juncan Electronic Equipment Co. Ltd., Shenzhen, China. GR with an average particle size less than 10 μm and a specific surface area of 260–350 m^2^ g^−1^ was supplied by Changzhou Sixth Element Material Technology Co. Ltd., Changzhou, China, and 2,5-dimethyl-2,5-bis(tert butylperoxy)hexane (DBPMH) was produced by Dongguan Caiyuan Silicone Co. Ltd., Dongguan, China.

### Preparation of the microwave-absorption composites

MPVQ/CIPs/GR composites were prepared by mechanical blending. First, MPVQ with a weight of 100 g was placed into a two-roll miller (HX-8106-6, Hongxiang Machinery Co. Ltd., Dongguan, China) for plastication. Then, silica, CIPs, GR and DBPMH with weights of 10, 250, 3 and 2 g, respectively, were added sequentially to obtain uniform compounds. Subsequently, the compounds were maintained at room temperature for 12 h and then vulcanized at 160 °C for 30 min to prepare vulcanized rubber sheets. Finally, the sheets were post-cured at 180 °C for 2 h in an oven (PH-303A, Yiheng Co. Ltd., Shanghai, China).

### Testing and characterization

#### Crosslinking density

The crosslinking density of the composites was measured by the equilibrium swelling method. One gram of samples with a thickness of 1.5 mm was immersed in toluene (the volume ratio of the sample to toluene was 1:100) in a reagent bottle at room temperature for 72 h to achieve swelling equilibrium. The samples were removed and dried with filter paper and then weighed.

Based on the Flory equation, the crosslinking density can be calculated by formula (1)^22,23^:

1 $$M_{c} = - \frac{{\rho_{0} V_{m} \varphi^{1/3} }}{{\ln (1 - \varphi ) + \varphi + \chi \varphi^{2} }}$$

where *ρ*_*0*_ is the density of the composites before swelling, *V*_*m*_ is the molar volume of the solvent, *φ* is the volume fraction of the rubber phase after swelling of the composite, and *χ* is the interaction parameter of the solvent and rubber, which is equal to 0.465 for toluene and silicone rubber.

#### Mechanical properties

The tensile properties of the cured samples were characterized by a universal material testing machine (UT-2080, Youken Co. Ltd., Guangzhou, China) according to the Chinese standard GB/T 528-2009 at a tensile speed of 500 mm min^−1^. Hardness was measured according to the Chinese standard of GB/T 531.1-2008 by a rubber hardness metre (LX-A, Liuling Co. Ltd., Shanghai, China).

#### Thermal-ageing performance

The thermal-ageing performance of the cured samples was tested in an oven (GT-7017-EL1, Gaotie Co. Ltd., Dongguang, China) according to the Chinese standard GB/T 3512-2014 at temperatures of 200, 220 and 240 °C for 24 h.

#### Morphology analysis

The fracture surface morphologies of the composites after thermal ageing at different temperatures were investigated under an optical microscope (BK-POL, Aote Co. Ltd., Chongqing, China) and a scanning electron microscope (EV018, Zeiss Co. Ltd., Germany). The samples were frozen with liquid nitrogen and then broken. The fractured surfaces of the samples were sputtered with gold and then observed for SEM analysis.

#### Attenuated total reflection-Fourier transform infrared spectrometry (ATR-FTIR) analysis

The ATR-FTIR spectra of specimens of the MPVQ/CIPs/GR composites were obtained with a resolution of 4 cm^−1^ in the range of 400–4000 cm^−1^ using a Nicolet spectrometer (VERTEX70, Bruker. Co. Ltd., Germany).

#### Thermogravimetric analysis (TGA)

Thermogravimetric analysis was performed at a heating rate of 20 °C min^−1^ in an air atmosphere with temperatures ranging from 35 to 900 °C using a thermal gravimetric analyser (TG209F3, NETZSCH. Co. Ltd., Germany).

#### X-ray diffraction (XRD) analysis

The X-ray diffraction pattern of the specimens was determined by an X-ray diffractometer (X'pert PRO, PANAlytical Analytical Instrument Co. Ltd., Netherlands) with Cu–Ka as the radiation source (λ = 0.154178 nm) at a step size of 0.033°, a test voltage of 40 kV and a current of 40 mA.

#### Microwave-absorption property measurements

The complex permittivity and permeability of the MPVQ/CIPs/GR composites in the frequency range of 0.2–18.0 GHz were measured by a vector network analyser (ZVB43, Rhodes & Schwartz Co. Ltd., Germany) according to coaxial-line theory. Concentric-ring samples with an inner diameter of 3.04 mm and an outer diameter of 7.00 mm were prepared by a puncher. In addition, thermally aged CIPs were uniformly mixed with fusible paraffin, with a mass ratio of the powders to paraffin of 5:2. Then, the mixtures were pressed into a cylindrical mould to obtain similar concentric rings.

## Results and discussion

### Mechanical properties and crosslinking densities

**Figure 1:**
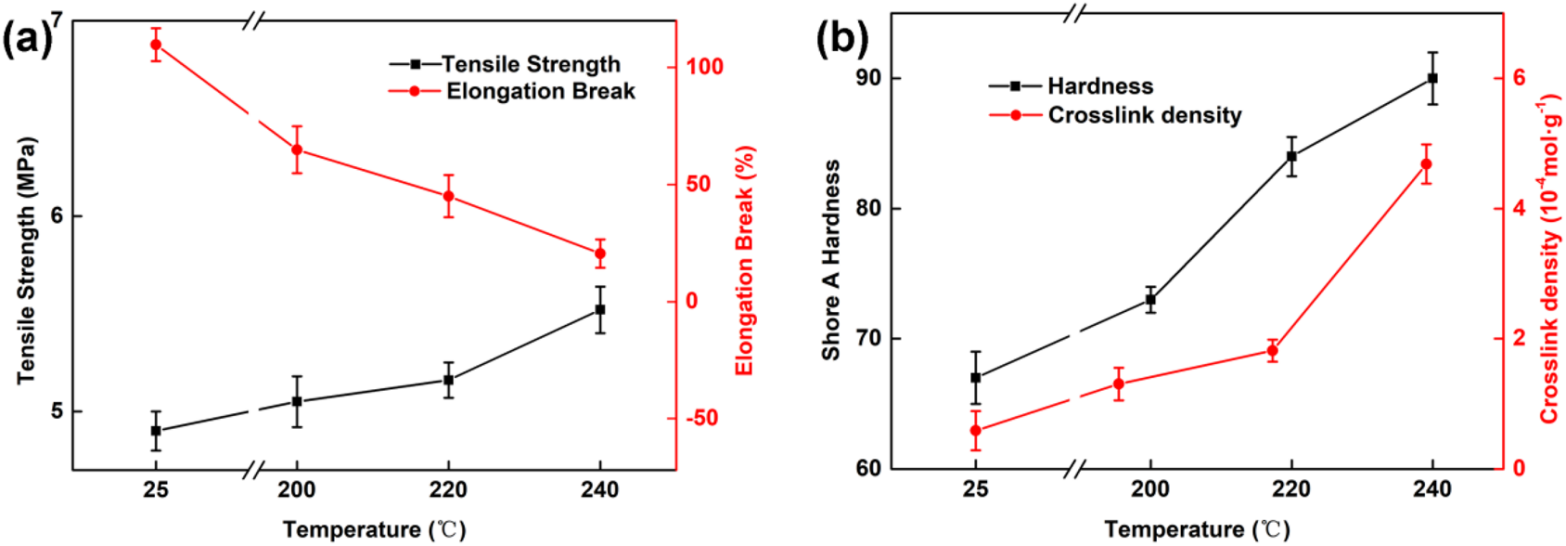
The tensile strength and elongation at break (**a**) and hardness and crosslink density (**b**) of the composites under different ageing temperatures.

Figure 1 illustrates the effects of thermal-ageing temperature on the mechanical properties and crosslink density of the MPVQ/CIPs/GR composites. As shown in Fig. 1a, the tensile strength of the unaged vulcanizate is 4.90 MPa, which increases to 5.52 MPa after thermal ageing at 240 °C. The elongation at break of the unaged sample decreases from 109.74 to 20.56% (aged at 240 °C).

Figure 1b shows that the hardness and crosslink density of the samples increase from 67° and 5.88 × 10^−5^ mol g^−1^ to 90° and 4.69 × 10^−4^ mol g^−1^, respectively, after hot-air ageing at 240 °C. The results can be attributed to the enhanced crosslink density of the MPVQ rubber after thermal ageing^24–27^.

### Morphological and structural characteristics

**Figure 2:**
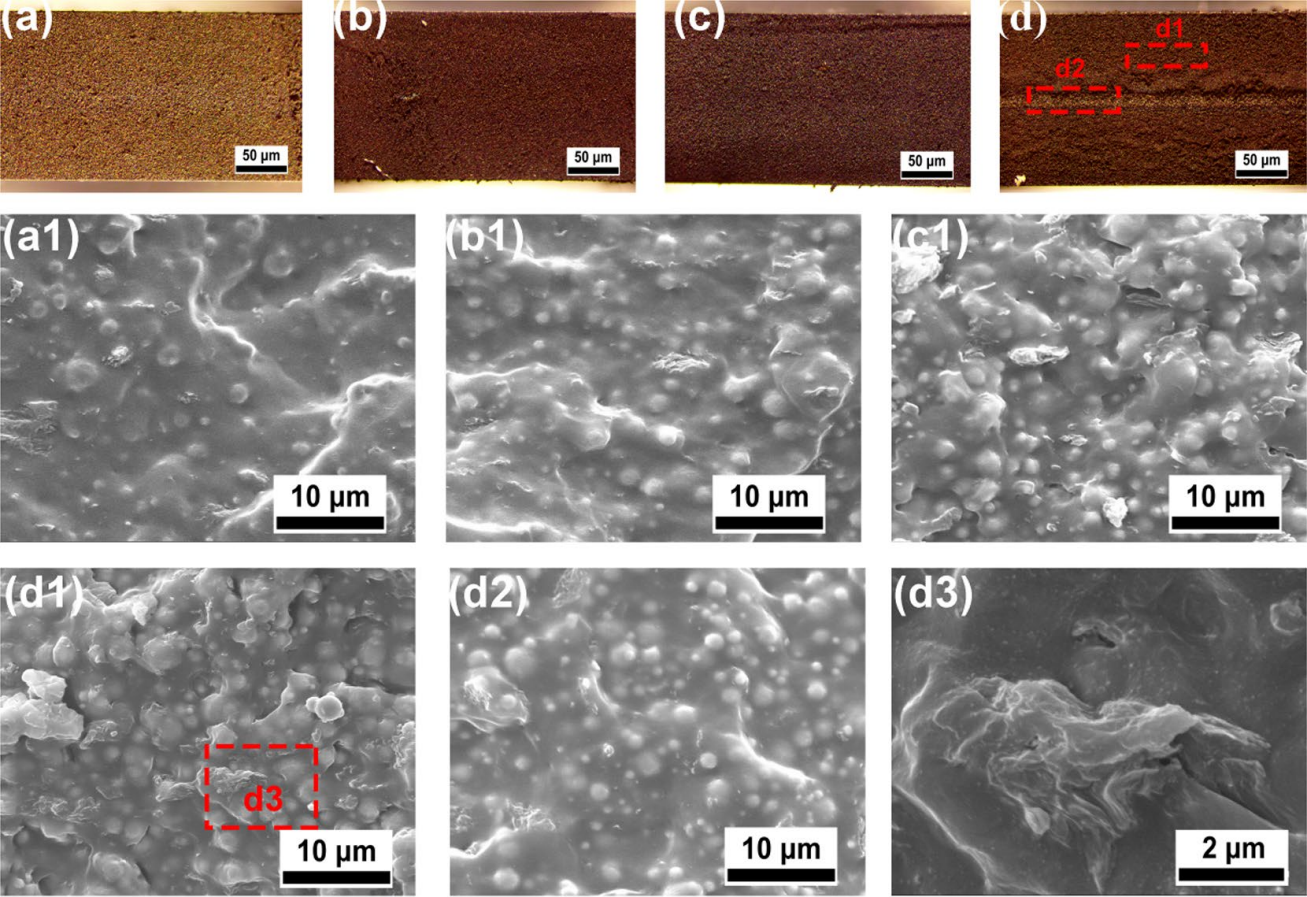
Optical microscopy and SEM images of the fractured surfaces of the composites after ageing at different temperatures: unaged (**a**, **a1**), 200 °C × 24 h (**b**, **b1**), 220 °C × 24 h (**c**, **c1**), 240 °C × 24 h (**d**, **d1**, **d2**) and aggregated graphene (**d3**).

Optical microscopic images of the fractured surfaces (a–d) and SEM images of the fractured surfaces (a1, b1, c1, d1, d2, d3) are shown in Fig. 2. As shown in Fig. 2a–d, with increasing thermal-ageing temperature, the colour of the fractured surfaces of the samples gradually deepens. Furthermore, the overall colour of most samples is uniform except for the sample after ageing at 240 °C, which shows obvious delamination in the middle layer of the fractured surface. The colour of the middle layer of the fractured surface is light, which is similar to that of the unaged sample. However, the layers above and below the middle layer are dark.

The SEM images show that CIPs are uniformly dispersed in the MPVQ matrix, and the interface between them is blurred, indicating a strong interfacial interaction between the powders and the MPVQ matrix. With increasing ageing temperature from 200 to 240 °C, the gaps in the CIPs gradually become closer (Fig. 2a1–d1) because of the increased crosslinking density and CIPs aggregation during the thermal-ageing process. In addition, multilayered graphene sheets can be observed in Fig. 2d3. In addition, the distribution of CIPs in the d2 area (enlarged in Fig. 2d2) is more uniform than that in the d1 area (amplified in Fig. 2d1), indicating that the thermal-ageing degree of the middle layer of the sample after ageing at 240 °C is lower than that of the outer layers because thermal oxidation of the composites occurred from the external to the inner areas. Furthermore, more graphene aggregation is shown in Fig. 2d3. Due to the obvious fusion of the CIPs after ageing at 240 °C, a barrier is generated to prevent oxygen penetration into the interior of the composites.

### XRD analysis

**Figure 3:**
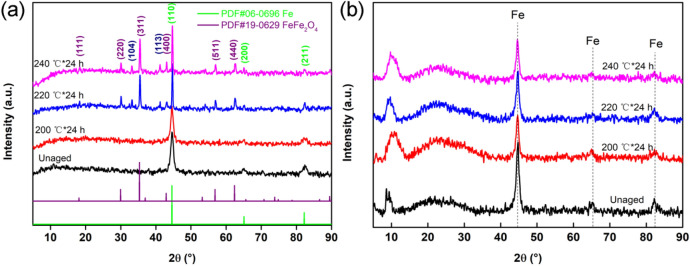
XRD curves of the composites aged at different temperatures: CIPs (**a**) and the MVPQ/CIPs/GR composites (**b**) before and after thermal ageing.

The XRD patterns of CIPs (Fig. 3a) and the MVPQ/CIPs/GR composites (Fig. 3b) aged at different temperatures are depicted in Fig. 3.

As shown in Fig. 3a, three obvious diffraction peaks located at 44.67°, 65.02° and 82.33° are ascribed to the (110), (200) and (211) crystal planes of Fe (PDF#06-0696), respectively. In addition, the diffraction peaks at 18.27°, 30.09°, 35.42°, 43.05°, 56.94° and 62.51° are ascribed to the (111), (220), (311), (400), (511) and (440) lattice planes of Fe_3_O_4_ (PDF#19-0629), respectively. Apart from the diffraction peaks of Fe and Fe_3_O_4_, two other weak peaks located at 33.24° and 41.16° are ascribed to the (104) and (113) crystal planes of Fe_2_O_3_ (PDF#33-0664), respectively, indicating that the pristine CIPs are mainly oxidized into Fe_3_O_4_ in the hot-air environment. In Fig. 3b, as the thermal-ageing temperature increases, the peaks of Fe become slightly weak, and the two broad peaks located from 6° to 30° corresponding to the rubber matrix are enhanced. However, no obvious diffraction peaks of Fe_3_O_4_ appeared in the spectra, indicating that only a small amount of CIPs had oxidized to Fe_3_O_4_ because of the protection of the rubber matrix from the CIPs.

### ATR-FTIR analysis

**Figure 4:**
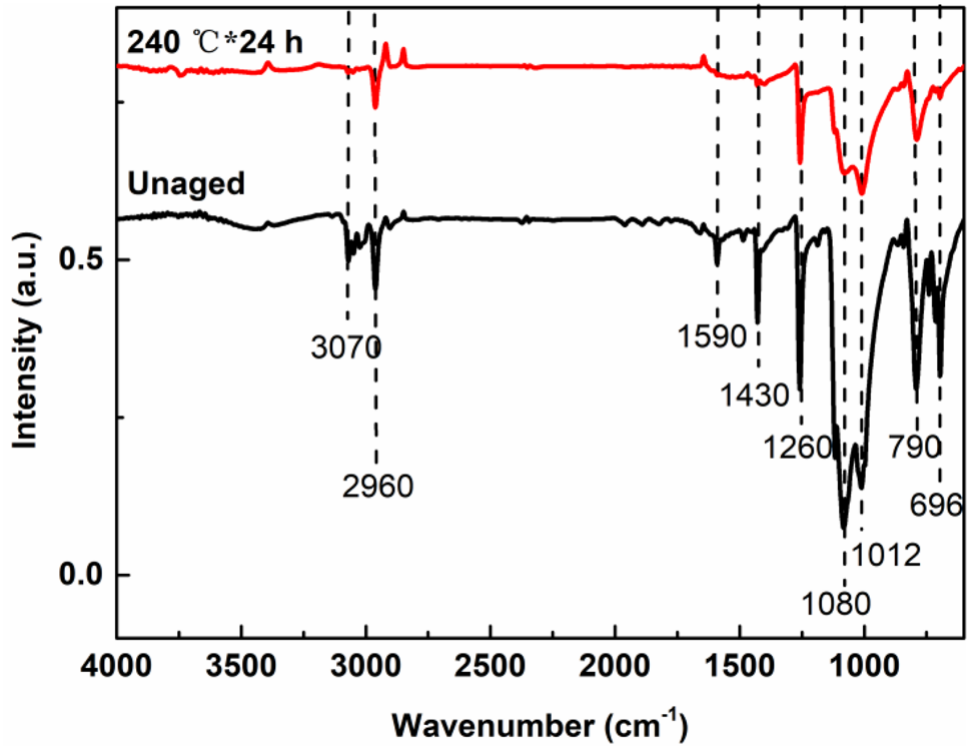
ATR-FTIR spectra of the MVPQ/CIPs/GR composites before and after thermal ageing at 240 °C for 24 h.

Figure 4 shows the ATR-FTIR spectra of the MVPQ/CIPs/GR composites before and after thermal ageing at 240 °C for 24 h.

As shown in Fig. 4, two absorption peaks are located at 1012 and 1080 cm^−1^, which are ascribed to the stretching vibration of Si–O–Si groups^28^. The peak appearing at 1590 cm^−1^ is associated with the C=C stretching vibration. The characteristic peaks at 1430 and 3070 cm^−1^ are attributed to the C–Si and C–H stretching vibrations of phenyl, respectively^29^. The peak at 2960 cm^−1^ originates from the C–H symmetric stretching vibration in the methyl group (–CH_3_). The peaks at 1260, 790 and 696 cm^−1^ are assigned to the symmetric deformation vibration and asymmetric and symmetric stretching vibrations of –CH_3_ in the Si–(CH_3_)_2_ groups, respectively^30^. After ageing at 240 °C, the intensity of the characteristic peaks of the Si–O–Si and Si–(CH_3_)_2_ groups became weak, and the peaks of C=C and phenyl nearly disappeared, indicating thermal degradation of the MVPQ matrix.

### TGA

**Figure 5:**
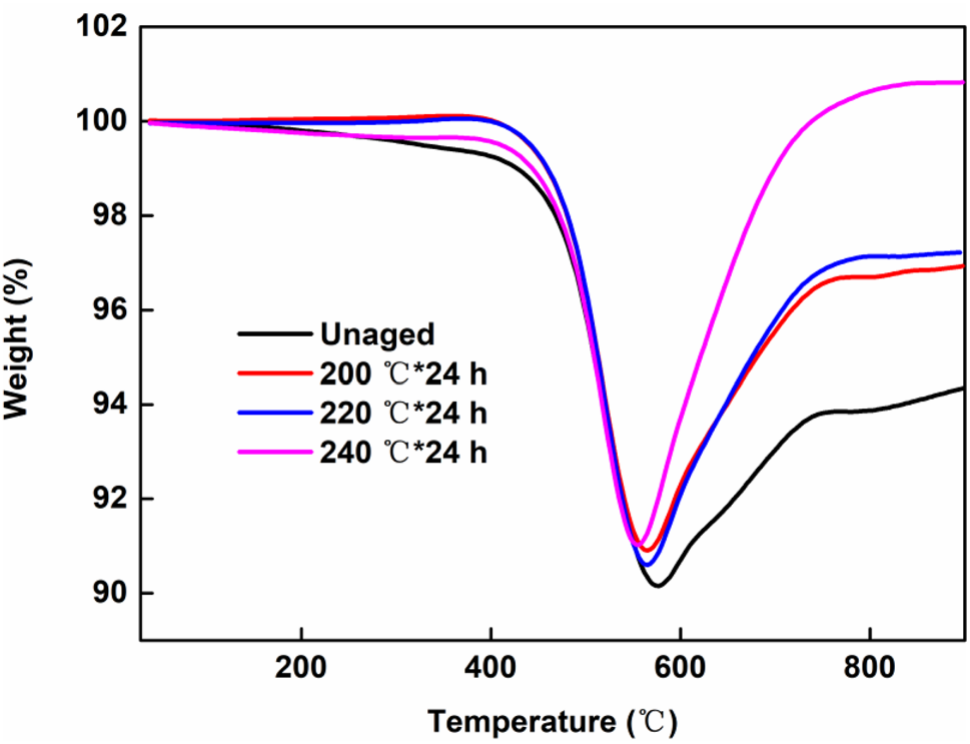
TGA curves of the unaged and aged MVPQ/CIPs/GR composites with ageing temperatures of 200, 220 and 240 °C for 24 h.

TGA curves of the unaged and aged composites with different ageing temperatures are shown in Fig. 5.

As compared to the unaged sample with an initial degradation temperature of 162 °C, the samples aged at 200 and 220 °C show initial degradation temperatures at 381 and 387 °C respectively because of the increased crosslinking densities during thermal-ageing. Thus, the diffusion of oxygen was prohibited, and the thermal stability of the composites was enhanced to some extent. However, when the aging temperature reached 240 °C, the rubber matrix was seriously degraded, leading to a decrement of the initial degradation temperature down to 167 °C, indicating the decline of the thermal stability of the composites. When the temperature is higher than 570 °C, the mass increment of all the samples is mainly ascribed to the oxidation of CIPs. In addition, because thermal-oxygen degradation of the MVPQ matrix results in an increase of the CIPs concentration. Consequently, the residual mass of the composites after oxidation at 900 °C increases with elevated ageing temperature.

### Microwave-absorption properties

**Figure 6:**
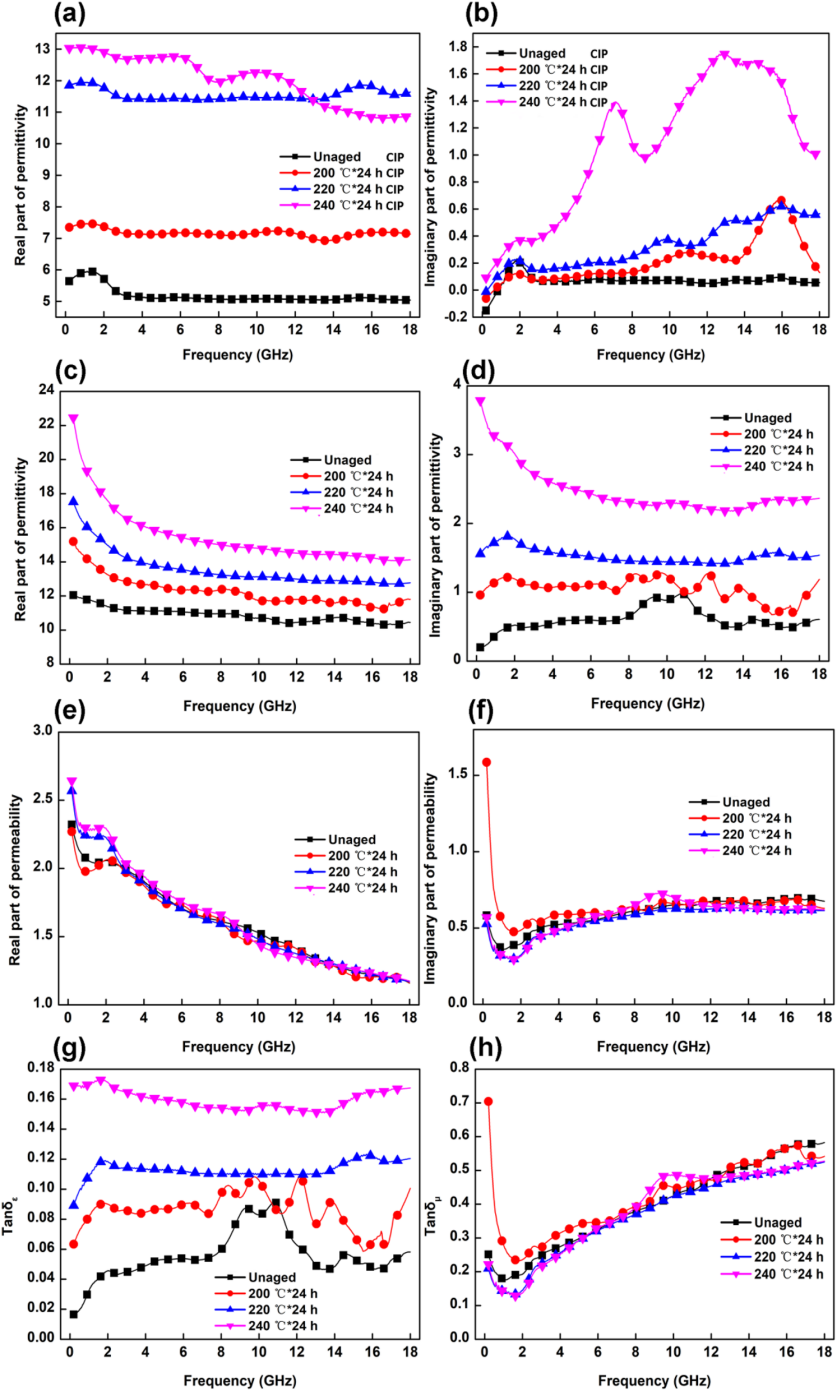
*ε*′ (**a**) and *ε*″ (**b**) values of CIPs aged at the different temperatures; electromagnetic parameters of the composites aged at the different temperatures: *ε*′ (**c**), *ε*″ (**d**), *μ*′ (**e**), *μ*″ (**f**), tan*δ*_*ε*_ (**g**), and tan*δ*_*μ*_ (**h**).

Based on electromagnetic theory, *ε*′ and *ε*″ indicate the storage capacity of electric energy and magnetic energy, respectively, and *μ*′ and *μ*″ represent the loss capacity of electric energy and magnetic energy, respectively^31^. To investigate the electromagnetic absorption properties of composites, the electromagnetic parameters are shown in Fig. 6.

The relative complex permittivity of the CIPs after oxidation is shown in Fig. 6a,b. The *ε*′ and *ε*″ values of CIPs increase with increasing thermal-ageing temperature, which is attributed to CIPs oxidation to Fe_3_O_4_. According to the free electron theory^32,33^:

2 $$\varepsilon^{\prime\prime} = \sigma /(2\pi \varepsilon_{0} f)$$

where *σ* is the conductivity, *ε*_*0*_ is the free space permittivity (8.854 × 10^12^ F m^−1^). It can be seen from Eq. (2) that a large value of *σ* induces a high *ε*″ value, indicating that the CIPs after oxidization to Fe_3_O_4_ show better conductive performance. In the crystal structures of Fe_3_O_4_, Fe^2+^ and Fe^3+^ ions show a random distribution in their octahedral position, and the electrons can be quickly transferred between the two valence states of iron ions to achieve excellent conductivity^34^.

As shown in Fig. 6c,d, the *ε*′ and *ε*″ values of the composites show a decreasing trend as the frequency increases from 0.2 to 18.0 GHz. Such a phenomenon may occur in many carbon nanomaterials, such as graphene and carbon nanotubes, which is named frequency-dispersion behaviour^35^. This behaviour significantly improves *ε*′ and *ε*″ values in the frequency range from 0.2 to 18.0 GHz with increasing thermal-ageing temperature, which is ascribed to the following two aspects. First, CIPs aggregation is beneficial to the formation of more conductive pathways. Second, some of the CIPs are oxidized to Fe_3_O_4_ during the thermal-ageing process, showing better conductive performance. The *ε*″ values of the unaged sample and the samples aged at 200 °C (in Fig. 6d) show dramatic fluctuations, and several resonant peaks appear in the frequency range because more voids and defects exist in the composites at lower temperatures, resulting in the accumulation of charges and further enhancement of interface polarization, dipole polarization and related relaxation^36,37^.

The real part (*μ*′) and imaginary part (*μ*″) of the permeability of the samples are shown in Fig. 6e,f, respectively. With increasing ageing temperature, the *μ*′ and *μ*″ values show no obvious variation due to the low oxidation degree of CIPs. Furthermore, the dielectric loss tangent (tan*δ*_*ε*_ = *ε*″/*ε*′) and the magnetic loss tangent (tan*δ*_*μ*_ = *μ*″/*μ*′) indicate the absorption capacity of the sample. In Fig. 6g,h, the tan*δ*_*μ*_ of each sample is higher than the tan*δ*_*ε*_, indicating that magnetic loss plays a key role in the process of electromagnetic-wave absorption.

According to the transmit line theory, the reflection loss (*RL*) value of composites can be calculated using the relative complex permeability and permittivity at a given frequency and absorber thickness. The calculation equations are as follows^38,39^:

3 $$RL = {\text{20log}}_{10} \left| {\frac{{Z_{in} - Z_{0} }}{{Z_{in} + Z_{0} }}} \right|$$

where *Z*_*0*_ is the free-space impedance, *Z*_*in*_ is the normalized input impedance, and the corresponding calculation is shown in formula (4):

4 $$Z_{in} = \sqrt {\frac{{\mu_{r} }}{{\varepsilon_{r} }}} \tanh \left[ {j\left( {\frac{2\pi fd}{c}} \right)\sqrt {\mu_{r} \varepsilon_{r} } } \right]$$

where *f* is the frequency of the electromagnetic wave, *c* is the speed of light, and *d* is the thickness of the samples.

**Figure 7:**
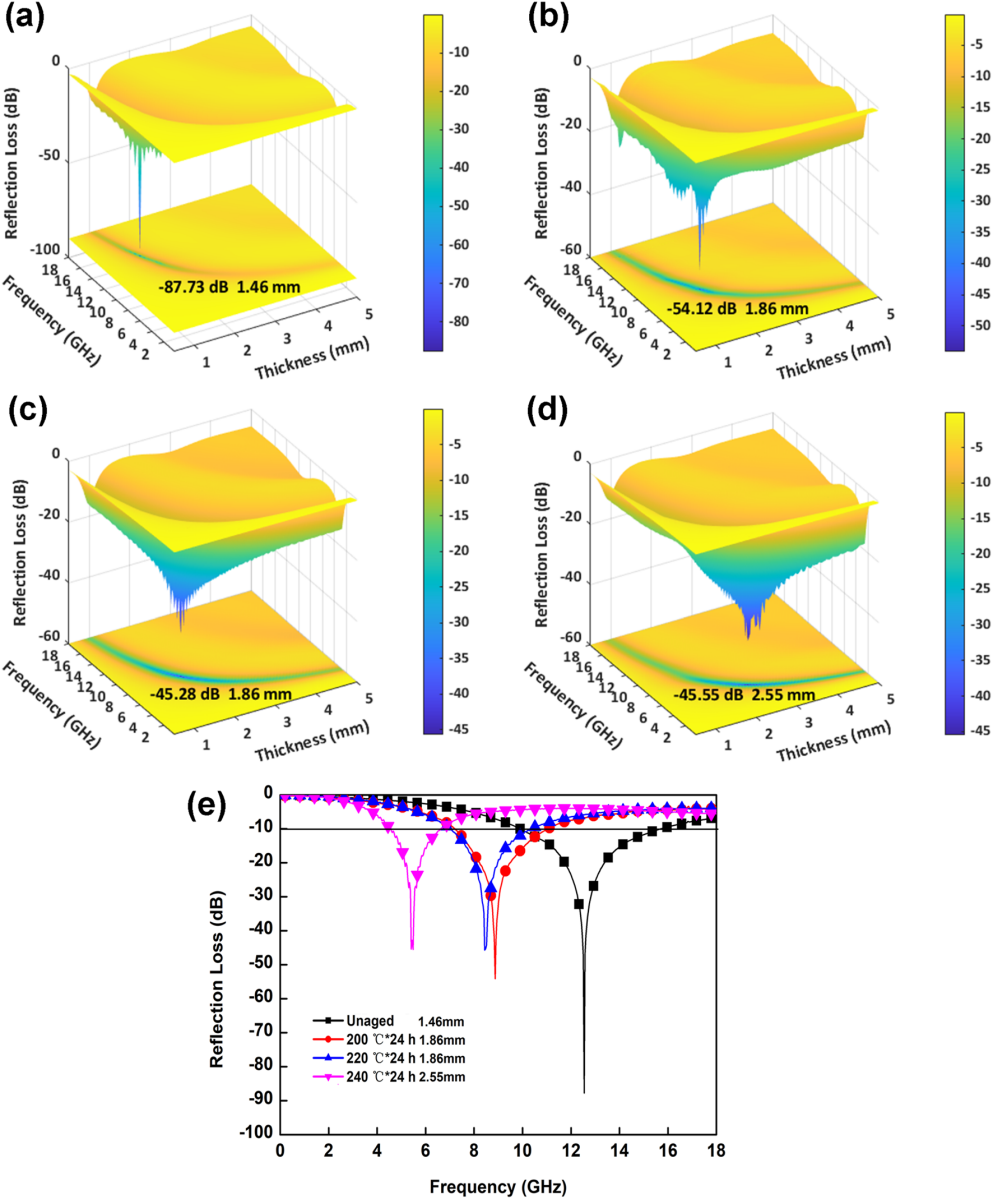
*RL* of the composites aged at different temperatures: unaged (**a**), 200 °C × 24 h (**b**), 220 °C × 24 h (**c**), and 240 °C × 24 h (**d**) and the influence of the frequency on the *RL*_*min*_ (**e**).

The effect of the frequency on the reflection loss (*RL*) of the composites after ageing at different temperatures is shown in Fig. 7. The unaged sample has the lowest *RL*_*min*_ of − 87.73 dB and a minimum thickness of 1.46 mm at 12.54 GHz with a widest *EAB* of 5.8 GHz (9.9–15.7 GHz) compared with the other composites. After ageing at 200 °C, the composites show a *RL*_*min*_ of − 54.12 dB with a thickness of 1.86 mm at 8.87 GHz, and the *EAB* reaches 3.8 GHz (7.2–11.0 GHz). However, when the ageing temperature is 240 °C, the *RL*_*min*_ reaches − 45.55 dB at 5.48 GHz with a thickness of 2.55 mm, and the corresponding *EAB* is 2.0 GHz (4.6–6.6 GHz), indicating that the microwave-absorption properties decrease with increasing thermal-ageing temperature. Meanwhile, the absorption peaks corresponding to the *RL*_*min*_ shift towards the low-frequency region.

Magnetic loss is theoretically derived from natural magnetic resonance, the eddy current effect and magnetic hysteresis^40^. Natural magnetic resonance occurs in the low-frequency region, while the resonance in the high-frequency region is exchange resonance. The eddy current coefficient *C*_*0*_ was applied to analyse magnetic loss, which can be described by formula (5)^41,42^:

5 $$C_{0} = \mu^{\prime\prime}(\mu^{\prime})^{ - 2} f^{ - 1} = 2\pi \mu_{0} d^{2} \delta$$

where *δ* represents electrical conductivity and *μ*_*0*_ is the vacuum permeability. If *C*_*0*_ remains unchanged, eddy current loss is the main mode of magnetic loss.

**Figure 8:**
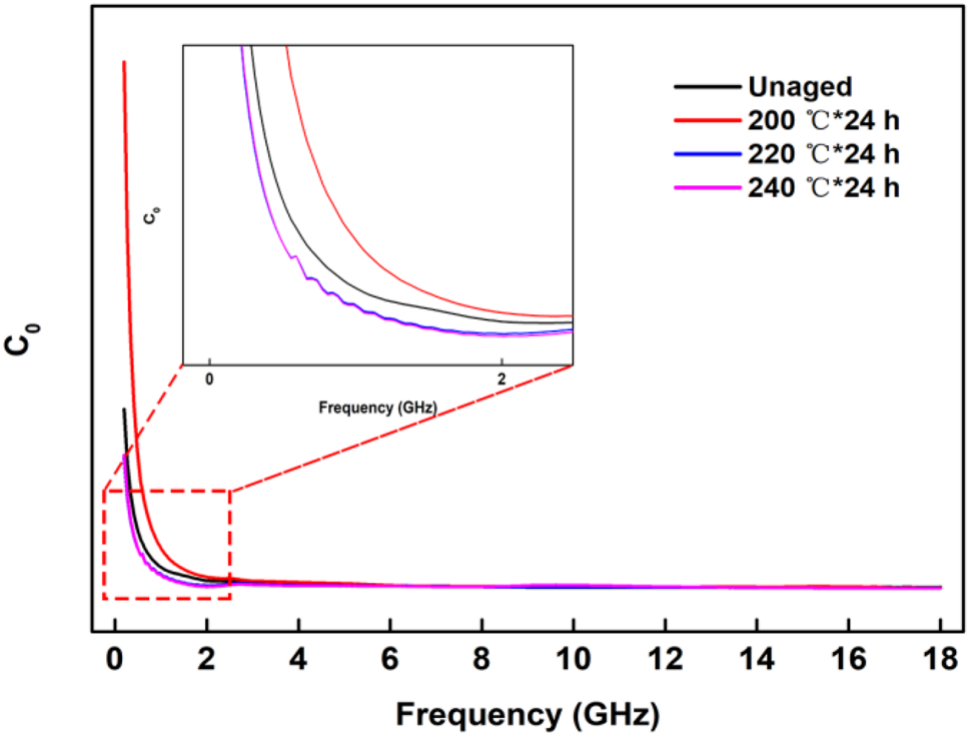
*C*_*0*_-*f* curves of the MPVQ/CIPs/GR composites.

In Fig. 8, the natural resonance of the composite aged at 200 °C is much stronger in the frequency range of 0.2–2.5 GHz because more defects and reactive groups are present at the lower thermal-ageing temperature. The *C*_*0*_ values of all the samples at 2.5–18.0 GHz remain constant, indicating that eddy current loss plays a key role in the magnetic loss of the microwave absorbers.

To further analyse the microwave-absorption properties of the composites, the attenuation constant of the samples was applied, which was calculated by formula (6)^43,44^:

6 $$\alpha = \frac{\sqrt 2 \pi f}{c} \times \sqrt {(\mu^{\prime\prime}\varepsilon^{\prime\prime} - \mu^{\prime}\varepsilon^{\prime}) + \sqrt {(\mu^{\prime}\varepsilon^{\prime\prime} + \mu^{\prime\prime}\varepsilon^{\prime})^{2} + (\mu^{\prime\prime}\varepsilon^{\prime\prime} - \mu^{\prime}\varepsilon^{\prime})^{2} } }$$

**Figure 9:**
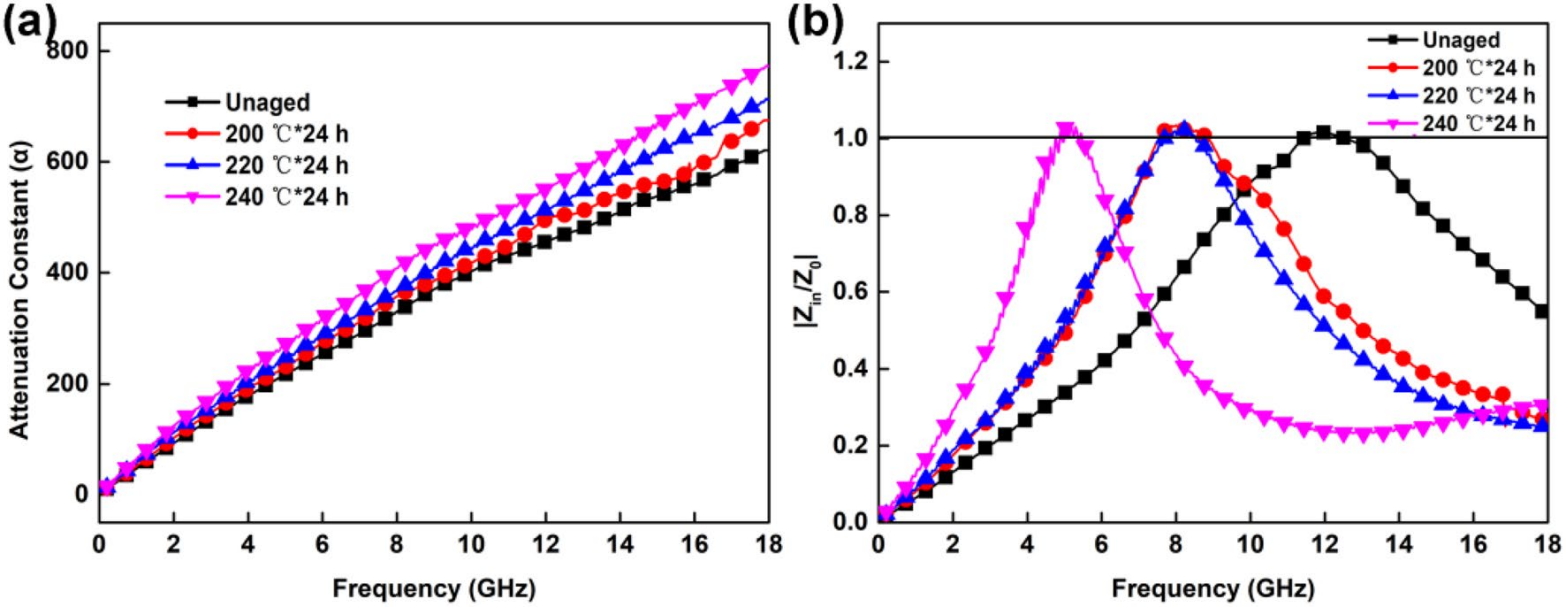
The attenuation constant (**a**) and |*Z*_*in*_/*Z*_*0*_| (**b**) of the composites aged at different temperatures.

The effect of the frequency on the attenuation constant is shown in Fig. 9a; the attenuation constant of all the samples aged at different temperatures increases with increasing frequency, indicating that the samples have a superior attenuation ability over the electromagnetic wave in the frequency bandwidth ranging from 0.2 to 18.0 GHz. Furthermore, the higher thermal-ageing temperature will result in a larger attenuation constant, which is beneficial for enhancing dielectric loss.

In addition, impedance matching is important to electromagnetic-wave-absorption performance. |*Z*_*in*_/*Z*_*0*_| values corresponding to impedance matching are shown in Fig. 9b. When the |*Z*_*in*_/*Z*_*0*_| value is close to 1, most of the electromagnetic wave will enter the composites, and the reflection of the electromagnetic wave is reduced to the minimum. The |*Z*_*in*_/*Z*_*0*_| value for the unaged sample clearly has a larger scope, which is close to 1, indicating better impedance matching.

To compare the microwave-absorption properties of our prepared composites and the other ones filled with CIPs as the microwave absorber, some microwave-absorption properties of them were listed in Table 1. As seen from Table 1, our composites unaged and aged at 200 °C show excellent microwave-absorption performance in terms of *EAB*, matching thickness and the *RL*_*min*_. Considering the simple preparation method, it can be concluded that the MPVQ/CIPs/GR composites will become competitive candidates for high-efficiency microwave-absorption materials applied at an elevated temperature.

**Table 1:** The comparison of the MPVQ/CIPs/GR composites with other microwave-absorption composites incorporated with CIPs.

Microwave absorbing material	Matrix	*RL*_*min*_ (dB)	*EAB RL* < − 10 dB (GHz)	*f *(GHz)	Thickness (mm)	Refs.
CIP (45 vol%) MWCNTs (0.5 wt%)	Silicone rubber	− 35.00	2.9 (2.7–4.6)	3.50	2.0	^14^
CIPs (60 wt%) Graphene (5 wt%)	Polyurethane	− 30.10	7.1 (10.9–18.0)	15.60	1.0	^15^
CIPs (50 vol%) MWCNTs (0.5 vol%)	Epoxy-silicone resin	− 16.90	14.6 (3.4–18.0)	10.50	1.50	^45^
CIPs (41.5 wt%) ZnO (2.5 wt%) Graphene (6 wt%)	Paraffin	− 45.57	0.41 (0.3–0.71)	0.48	4.00	^46^
Poly-vinyl alcohol coated CIPs (50 wt%) Reduced graphene oxide (0.05 wt%)	Paraffin	− 29.89	8.4 (9.6–18.0)	14.88	2.50	^47^
CIPs/Ce_2_Co_16.6_Ni_0.4_	Paraffin	− 30.33	4.64 (8.70–13.34)	10.56	1.60	^48^
CIPs/Fe_3_O_4_ (45 wt%) Ti_3_C_2_T_X_ (5 wt%)	Paraffin	− 52.10	3.32 (7.20–10.52)	9.16	2.20	^49^
CIPs (50 vol%) Graphene (0.82 wt%)	Phenyl silicone rubber	− 87.73	5.8 (9.9–15.7)	12.54	1.46	This work (unaged)
		− 54.12	3.8 (7.2–11.0)	8.87	1.86	This work (200 °C)

**Figure 10:**
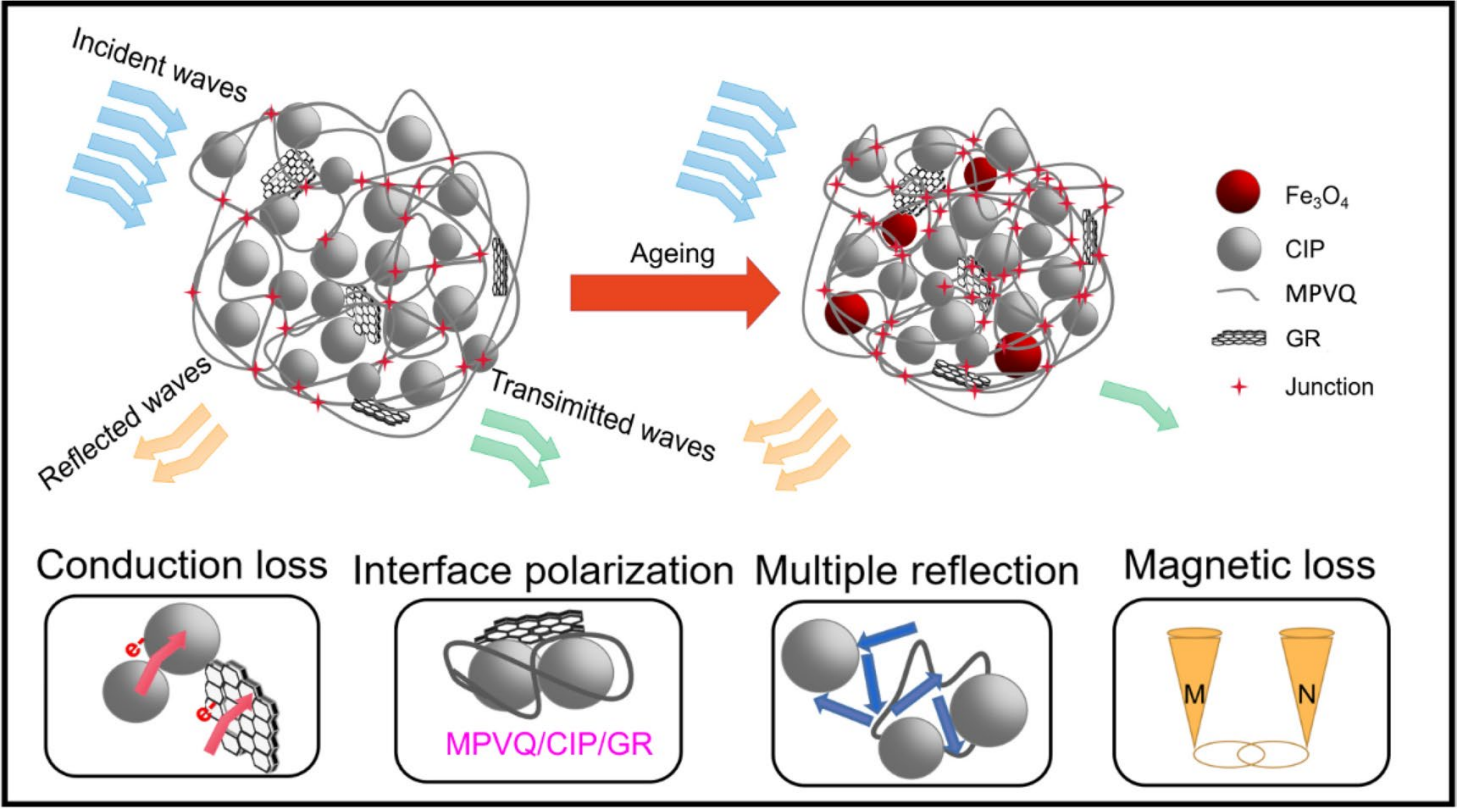
Schematic diagram of the influence mechanism of thermal-ageing on the electromagnetic wave-absorbing property of the composites.

The possible microwave-absorption mechanism of the thermal-ageing composites is shown in Fig. 10.

Firstly, CIPs and GR have an abundance of free electrons to generate microcurrent and electrically conductive networks, which contributes to enhancing conductive loss^50^. After thermal ageing, enrichment of the CIPs and the appearance of Fe_3_O_4_ further enhance the conductive loss. Secondly, multiple interfaces and some structural defects existed in the composites, leading to the multiple scattering and the reflection of the microwave, which extends the microwave-propagation path and further helps absorb more microwave^51–53^. Third, structural defects can act as polarization centres to improve dipole polarization and interface polarization^54–56^. However, some compact structures of the composites were generated because of thermal ageing, resulting in reduced interface polarization. Ultimately, magnetic loss derives from CIPs via natural low-frequency resonance, exchange resonance or the eddy current effect. Due to effective protection of the rubber matrix from CIPs, CIPs with minor oxidization rarely have any influence on magnetic loss. Considering the four explanations above, the enhanced thermal-ageing temperature caused excessive conduction loss, thus leading to impedance mismatching, which is the key to reducing the microwave-absorption performance of the composites as a whole^57^.

## Conclusions

In summary, MPVQ/CIPs/GR composites were prepared by mechanical blending. The influence of thermal-ageing temperature on microwave-absorption performance was investigated. The unaged composite exhibited superior microwave-absorption properties with a lowest *RL*_*min*_ of − 87.73 dB, a widest *EAB* of 5.8 GHz (9.9–15.7 GHz) and a minimum thickness of 1.46 mm at a frequency of 12.54 GHz. Outstanding microwave absorption properties were maintained with a *RL*_*min*_ of − 54.12 dB after thermal-ageing at 200 °C for 24 h because of minor oxidation of the composites. However, after ageing at 240 °C for 24 h, the *RL*_*min*_ reached − 45.55 dB at a frequency of 5.48 GHz with a thickness of 2.55 mm, and the *EAB* reached 2.0 GHz (4.6–6.6 GHz). Because of the concentration of CIPs in the rubber matrix and their partial oxidization to Fe_3_O_4_ particles, conduction loss significantly increased, resulting in impedance mismatching of the composites. Consequently, the composites exhibit excellent microwave-absorption performance in an elevated temperature below 200 °C.
